# Genome-wide analysis of tandem duplicated genes and their expression under salt stress in seashore paspalum

**DOI:** 10.3389/fpls.2022.971999

**Published:** 2022-09-30

**Authors:** Xu Hu, Jiangshan Hao, Ling Pan, Tao Xu, Longzhou Ren, Yu Chen, Minqiang Tang, Li Liao, Zhiyong Wang

**Affiliations:** ^1^College of Tropical Crops, Hainan University, Haikou, China; ^2^Key Laboratory of Genetics and Germplasm Innovation of Tropical Special Forest Trees and Ornamental Plants, Ministry of Education, College of Forestry, Hainan University, Haikou, China; ^3^School of Agriculture, Jinhua Polytechnic, Jinhua, China; ^4^College of Agro-Grassland Science, Nanjing Agricultural University, Nanjing, China

**Keywords:** abiotic stress, salt, seashore paspalum, tandem duplicated genes, transcriptome

## Abstract

Seashore paspalum (*Paspalum vaginatum*) is a halophytic, warm-season grass which is closely related to various grain crops. Gene duplication plays an important role in plant evolution, conferring significant plant adaptation at the genomic level. Here, we identified 2,542 tandem duplicated genes (TDGs) in the *P. vaginatum* genome and estimated the divergence time of pairs of TDGs based on synonymous substitution rates (Ks). Expression of *P. vaginatum* TDGs resulted in enrichment in many GO terms and KEGG pathways when compared to four other closely-related species. The GO terms included: “ion transmembrane transporter activity,” “anion transmembrane transporter activity” and “cation transmembrane transport,” and KEGG pathways included “ABC transport.” RNA-seq analysis of TDGs showed tissue-specific expression under salt stress, and we speculated that *P. vaginatum* leaves became adapted to salt stress in the earlier whole-genome duplication (WGD; ~83.3 million years ago; Ma), whereas the entire *P. vaginatum* plant acquired a large number of TDGs related to salt stress in the second WGD (~23.3 Ma). These results can be used as a reference resource to accelerate salt-resistance research in other grasses and crops.

## Introduction

High salinity is a major abiotic environmental stress that is reported to be responsible for reductions in plant growth and crop production worldwide (Roy et al., 2014; Kumari et al., 2015). The production of salt-tolerant crops is potentially a cost-effective approach to provide improved growth in saline soils (Rasheed et al., 2022). It is therefore of critical interest to unravel the salt-resistance mechanisms of halophytes and transfer these, if possible, to glycophytes (Kumari et al., 2015). Seashore paspalum (*Paspalum vaginatum* Sw.) is a halophytic, warm-season, perennial grass that has been utilized as turf for almost 100 years, especially in coastal and salt-affected regions across the world (Wu et al., 2018; Qi et al., 2019). *P. vaginatum* is one of the most saline-tolerant turfgrass species (Liu et al., 2011; Uddin et al., 2012; Spiekerman and Devos, 2020) and, as it is closely related to some of the world’s most important grain crops, including maize, sorghum and millet (Qi et al., 2019), may provide a gateway for cereal crop improvements in salt resistance.

Gene duplication is a fundamental process in genome evolution (Holland, 1999; Paterson et al., 2010), and can result from whole-genome duplication, tandem duplication, duplication mediated by transposable elements, segmental duplication, and/or retroduplication (Panchy et al., 2016; Guo et al., 2019; Qiao et al., 2019). Tandem duplication refers to the generation of tandem arrays consisting of identical sequences in close genomic proximity. This occurs due to unequal chromosomal recombination, a widespread phenomenon in plant genomes which plays significant evolutionary roles including in adaptation to changing environments (Cannon et al., 2004; Yu et al., 2015). Tandem duplication events have been implicated in various plant traits such as stress resistance and membrane function in arabidopsis and rice (Rizzon et al., 2006), disease resistance in Solanaceae and Brassicaceae species (Leister, 2004), signal transduction in legumes (Bellieny-Rabelo et al., 2013) and glucosinolate biosynthesis diversification in the mustard family (Hofberger et al., 2013).

However, little is known about tandem duplicated genes (TDGs) and their possible contributions to the genome evolution of salt-stress resistance in *P. vaginatum*. Here, we report a comprehensive study of genome-wide TDGs present in the genome of *P. vaginatum* and surmise their evolutionary contributions. The functions of the TDGs were proposed using GO and KEGG enrichment analyses. The use of RNA-seq data made it feasible to identify TDGs that respond to salt stress in various tissues. Our findings can provide further insights into *P. vaginatum* evolution, particularly in relation to salt resistance. In addition, this genetic resource might also be useful for salt resistance research into other grasses and crops.

## Materials and methods

### Data sources

Protein sequence and General Feature Format (GFF) files of *Oryza sativa*, *Setaria italica*, *Sorghum bicolor*, and *Zea mays* were downloaded from EnsemblPlants.^1^ We obtained a chromosome-level reference genome of diploid cultivated *P. vaginatum*: The reference genome size was 517.98 million bases (Mb), including 28,712 codable proteins. For genes with multiple transcripts, the longest transcript was selected for subsequent analysis.

### TDG analysis

TDGs were mainly identified based on protein sequences and the GFF file. Firstly, BLASTP (Altschul et al., 1997; settings: E < 1e–10; first 10 matches) was performed with protein sequences to search for all potential homologous gene pairs within each genome. Secondly, the blast results were analyzed in MCScanX (Wang et al., 2012) using a modified MCScan algorithm. Thirdly, duplicated gene pairs were identified using the downstream analysis tool (duplicate_gene_classifier) which is incorporated into the MCScanX package. Finally, duplicated gene pairs with code 3 (representing TDGs) were extracted. The non-synonymous (Ka) and synonymous substitution (Ks) frequencies of duplicated genes were calculated using ParaAT (Zhang et al., 2012), which was further used to compute the approximate dates of duplication and divergence events using the formula T = Ks/2λ, assuming a clocklike rate (λ) of 1.5 synonymous substitutions per 10^−8^ years (Yang et al., 2020; Wang et al., 2021b) for *P. vaginatum*. Moreover, the Ka/Ks ratio was also employed to show the selection pressure for the duplicated genes.

### GO term and KEGG pathway enrichment analyses

Based on the annotation information^2^ (Huerta-Cepas et al., 2017), TDGs from *P. vaginatum* were analyzed for GO term and KEGG pathway functional enrichment using the R package clusterProfiler (Wu et al., 2021). “Rich factor” is the ratio of the number of differentially-expressed genes annotated in a term (or pathway) to the number of all genes annotated in this term (or pathway).

### Identification and sequence analysis of the ABC gene family

The Hidden Markov Model (HMM) profile of the ABC domain (PF00664, PF00005, PF01061, PF02470 and PF01458) from the Pfam database^3^ (Mistry et al., 2021) was utilized to identify the ABC members in *P. vaginatum* by using the HMMER software (Mistry et al., 2013)(settings: E < 1e–10), and redundant sequences were removed manually. Additionally, all obtained ABC protein sequences were analyzed online using the CDD website^4^ (Marchler-Bauer et al., 2017) to verify conserved ABC domains. The members of the *P. vaginatum* ABC family were named according to chromosomal position. The number of amino acids, theoretical molecular weight (MW), and theoretical isoelectric point (pI) of the ABC family were obtained from the ExPASy web resource^5^ (Gasteiger et al., 2003). In order to explore the phylogenetic relationships of *P. vaginatum* ABC-family genes, data from 105 ABC proteins of *O. sativa* were downloaded for multiple sequence alignment using MAFFT (Rozewicki et al., 2019), and multiple sequences were trimmed using TBtools (Chen et al., 2020). Phylogenetic trees (ML) were constructed using IQ-TREE (Minh et al., 2020) (settings: -m MFP -bb 1000 -alrt 1000) and generated using iTol online tools^6^ (Letunic and Bork, 2021).

### RNA-seq and bioinformatics analysis

The *P. vaginatum* salt-resistant ecotypes USA17-18 were analyzed by RNA-seq. For the RNA-seq experiments, similar stolons were cultivated in plastic pots under typical conditions. Two-month-old plants were treated with 400 mM NaCl or water as a control for 8, 12, 24, or 48 h or 5 days before harvesting tissues. Total RNAs were isolated from leaves with three biological replicates at each stress stage. RNA-seq and *de novo* assembly Paired-end sequencing of cDNA libraries were performed using the HiSeq 2000 platform (Supplementary Table 10). Clean reads were aligned to the reference genome using HISAT2 (Kim et al., 2015). FPKM (fragments per kilobase per million mapped reads) was used to estimate the expression levels of individual genes (Supplementary Table 11). Differentially expressed genes (DEGs) were determined using the R package DESeq2 (Love et al., 2014) with a false discovery rate (FDR) of ≤ 0.05 and |log_2_ FC| ≥ 1 used as the threshold to determine statistically significant differences in gene expression. To determine whether these genes were tissue-specific to leaves or roots, gene expression patterns were compared in both transcriptomic data of control and treatment plants. Tissue-specifically expressed genes and co-expressed genes were defined based on the following rules: If the gene appeared up-regulated at least four times in one tissue but less than (or equal to) one time in another tissue, we considered it to be a tissue-specifically expressed gene; if the gene appeared up-regulated at least seven times in both tissues, it was considered to be a co-expressed gene.

## Results

### Identification and genomic distribution of TDGs

**Figure 1:**
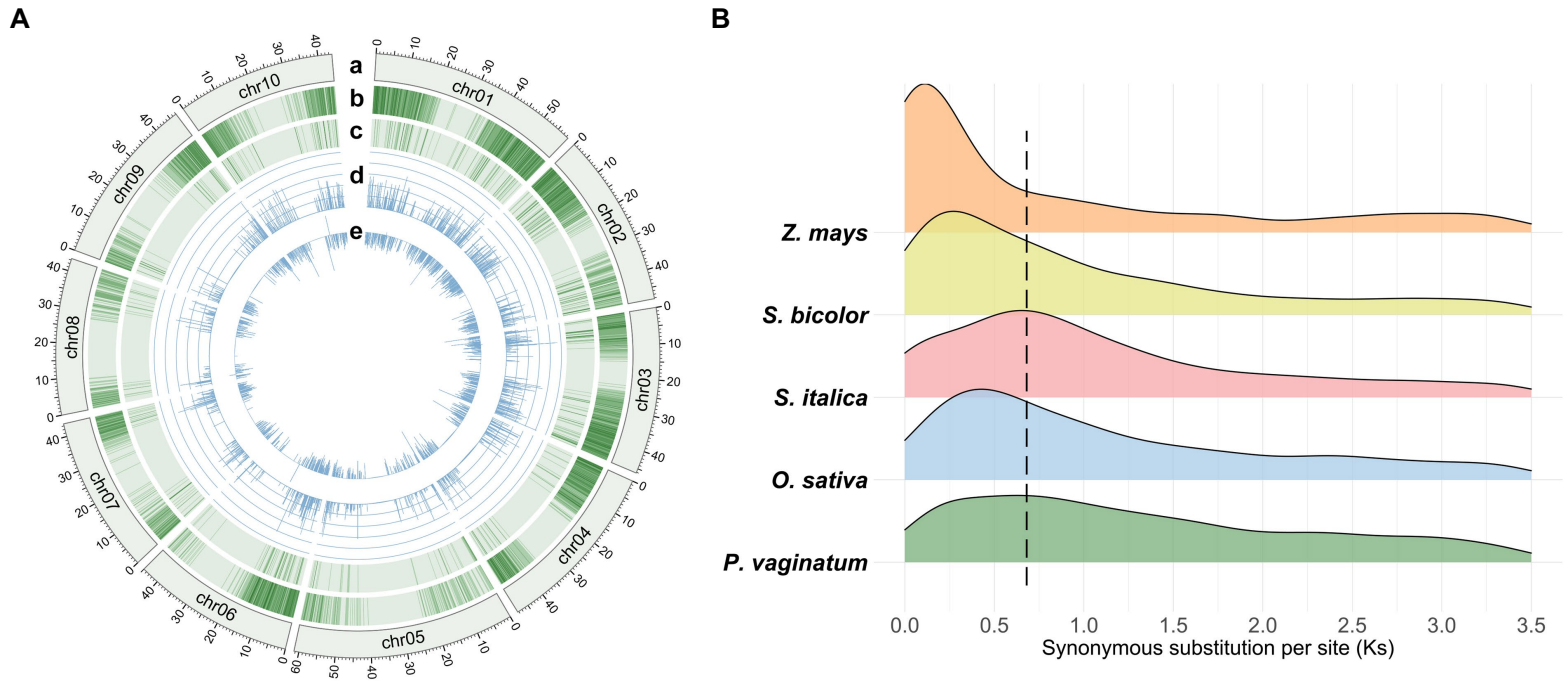
Overview of the *Paspalum vaginatum* genome and comparison of Ks. **(A)** Tracks from outer (a) to inner (e) rings indicate the following: (a) Chromosome size with units in Mb; (b) density of genes; (c) density of TDGs; (d) Ks of TDG pairs; (e) Ka/Ks values of TDG pairs and normalization by log2 (Ka/Ks). **(B)** Distribution of Ks calculated by TDG pairs between study species.

The *P. vaginatum* genome sequence consists of 517.98 Mb of DNA sequence and 28,712 protein coding genes. A total of 2,542 TDGs (8.85% of the gene set) were identified in the *P. vaginatum* genome, with a higher frequency than *O. sativa* (7.78%) and *Z. mays* (4.74%) but lower frequency than *S. italica* (11.55%) and *S. bicolor* (10.82%) (Supplementary Table 1). In addition, the distribution of TDGs was not uniform across chromosomes (Figure 1A): The most, 428, TDGs (16.84% of total TDGs), were located on chromosome 1 (chr01); the least, 134 (5.27% of the total TDGs) on chromosome 8 (chr08) (Supplementary Table 2). The synonymous substitution rates (Ks) in the TDG pairs were calculated for 1,581 gene pairs, and the distribution of Ks showed a single peak value ranging from 0.6 to 0.7 (Figure 1B; Supplementary Table 3). To infer the speciation occurrence time of *P. vaginatum*, the T = Ks/2λ method was used. Results indicated an estimated time of approximately 20.0–23.3 Ma, suggesting that *P. vaginatum* and *S. italica* might have shared common whole-genome duplicates. Ka/Ks values were then used to determine possible selection pressure between individual genes in a pair. The Ka/Ks values for *P. vaginatum* TDGs showed that there were few TDG pairs with Ka/Ks values much greater than 1, with most TDG pairs having Ka/Ks values much less than 1 (Supplementary Table 3), suggesting that most TDGs were under negative selection and that only a small number were under positive selection.

The number of TDGs in the same tandem cluster ranged from two to nine with the longest tandem clusters found with “UDP-glycosyltransferase” and “GRAS domain” (Table 1). We focused on TDGs with more than six genes in the same cluster and their functions (Supplementary Table 4). The majority of these long TDG clusters were found to be associated with salt resistance after reviewed evidence and included: “UDP-glycosyltransferase,” “GRAS domain,” “Auxin responsive protein,” “Cytochrome P450,” “FCS-type zinc-finger,” “Dirigent protein,” “BTB/POZ domain,” “Expansin, cellulose-binding-like domain,” “Glutathione S-transferase,” “RING-type zinc-finger” and “FAD-linked oxidoreductase” (Table 1).

**Table 1:** TDGs with more than six genes in the same cluster and their functions.

The number of TDGs in the same cluster	Chr	Description	Reviewed evidence
9	chr03	UDP-glycosyltransferase	Li et al. (2017); Wang et al. (2021a)
	chr05	GRAS domain	Wang et al. (2020); Zhang et al. (2020)
8	chr02	Auxin responsive protein	Bouzroud et al. (2018); Kang et al. (2018)
7	chr02	UDP-glucosyl transferase	Wang et al. (2021a,c)
	chr03	Cytochrome P450	Magwanga et al. (2019); Krishnamurthy et al. (2020)
	chr06	FCS-type zinc-finger	Qin et al. (2021)
	chr06	HIPP	--
	chr06	Dirigent protein	Wang et al. (2022)
6	chr01	BTB/POZ domain	Wan et al. (2019)
	chr01	Expansin, cellulose-binding-like domain	Chen et al. (2017)
	chr03	Nucleotide-diphospho-sugar transferase	-
	chr03	Glutathione S-transferase	Xu et al. (2015); Yang et al. (2019)
	chr04	RING-type zinc-finger	Jung et al. (2013); Agarwal and Khurana (2020)
	chr10	FAD-linked oxidoreductase	Zhao et al. (2021); Wu et al. (2022)

### The TDGs in *Paspalum vaginatum* contribute to adaptability, according to enrichment analyses

**Figure 2:**
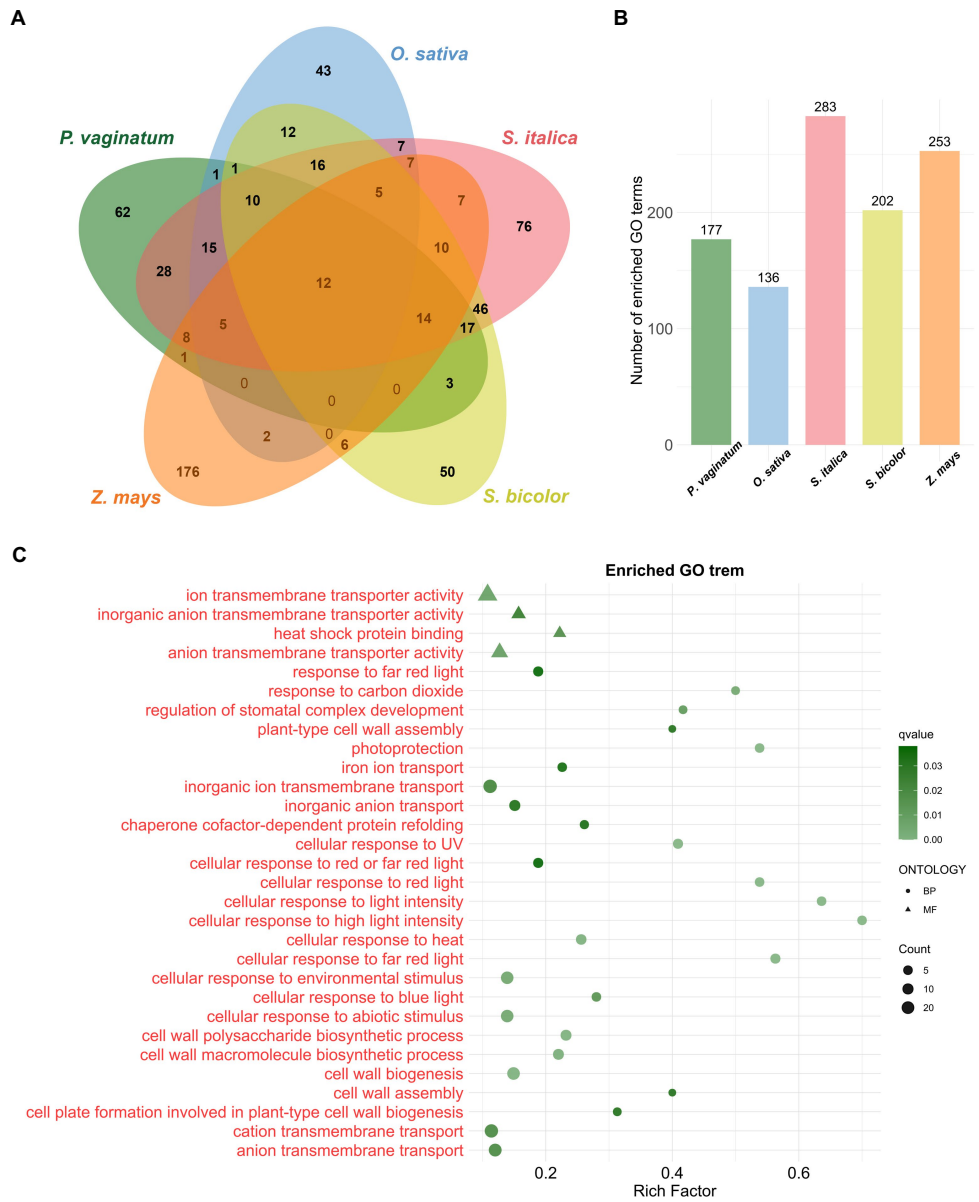
GO enrichment analysis of TDGs. **(A)** The venn diagram represents the shared and unique enriched GO terms among five species. **(B)** The size of enriched GO terms in each species. **(C)** The bubble diagram shows 30 significantly enriched GO terms. The terms in red are unique to *P. vaginatum* compared to the other four species.

To gain insights into the biological processes necessary for the adaptation to the environment, the 2,542 TDGs in *P. vaginatum* were analyzed for GO enrichment. The set of TDGs was involved in 177 significant biological processes. The maximum Rich Factor was 0.75 (with xyloglucan biosynthesis), followed by 0.70 (with cellular response to high light intensity; Figure 2B; Supplementary Table 5). Then, a comparative analysis of four grass species (*O. sativa*, *S. italica*, *S. bicolor*, and *Z. mays*) with *P. vaginatum* was performed. Based on the GO terms, the *P. vaginatum* TDGs were found to be enriched in 62 unique GO terms compared with other analyzed species (Figure 2A). These GO terms included “cellular response to light intensity,” “cellular response to UV” and “cellular response to heat” etc. (Figure 2C), which are related to adaptation to tropical climates. Other enriched GO terms included “ion transmembrane transporter activity,” “anion transmembrane transporter activity” and “cation transmembrane transport” etc. (Figure 2C), which might be associated with adaptation to a saline environment.

**Figure 3:**
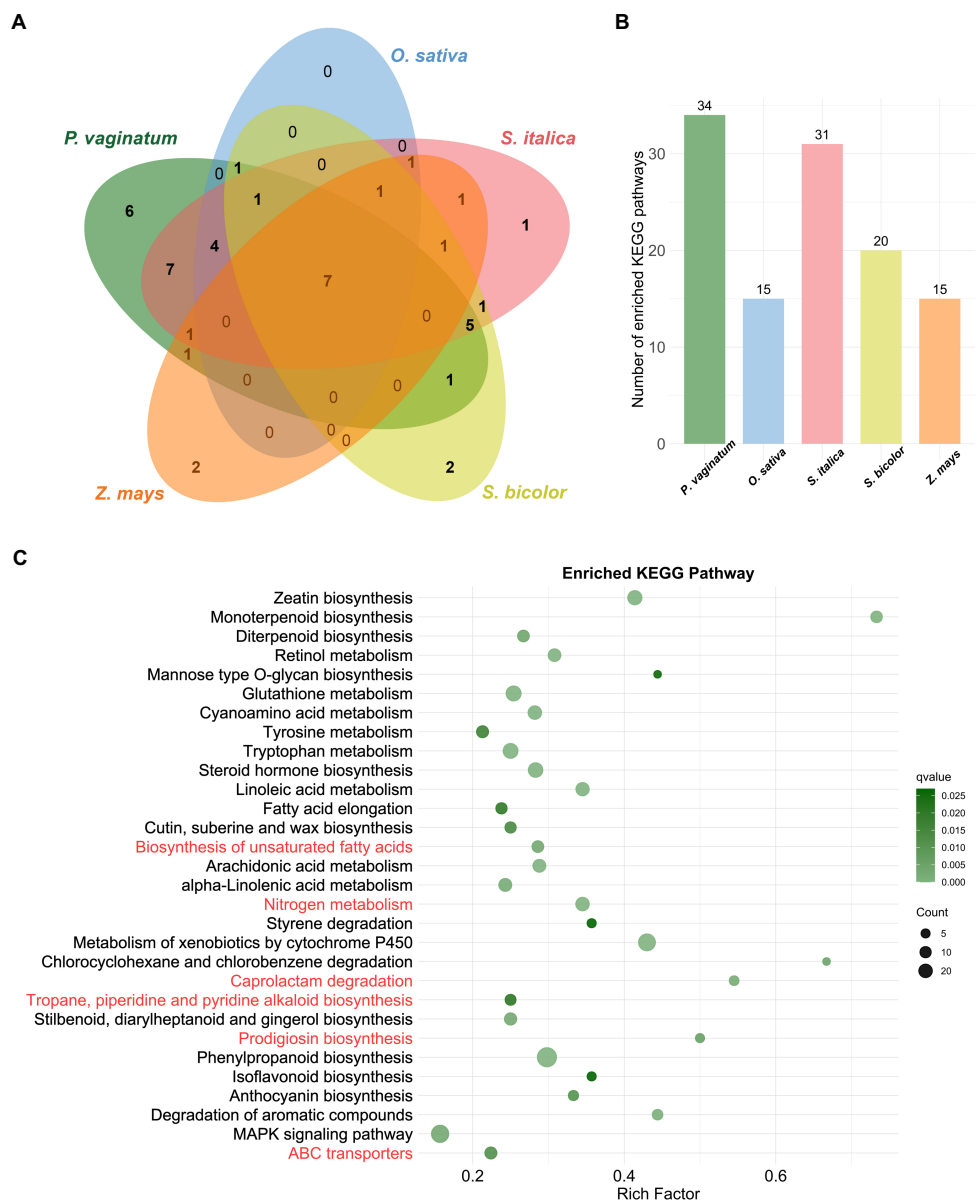
KEGG enrichment analysis of TDGs. **(A)** The venn diagram represents the shared and unique enriched KEGG pathways among five species. **(B)** The size of enriched KEGG pathways in each species. **(C)** The bubble diagram shows 30 significantly enriched KEGG pathways. The pathways in red are unique to *P. vaginatum* compared to the other four species.

KEGG pathway analysis provides classifications that are valuable for studying the complex biological functions of genes. Therefore, KEGG enrichment analysis was also performed on TDGs of the studied species. The results showed that TDGs in *P. vaginatum* were significantly enriched in 34 pathways (Figure 3B; Supplementary Table 6). The pathways enriched with the top three numbers of TDGs were “Phenylpropanoid biosynthesis,” “MAPK signaling pathway” and “Metabolism of xenobiotics by cytochrome P450.” In comparison to the other four species, *P. vaginatum* had six unique pathways (Figures 3A,C), five of which were related to KEGG pathway Metabolism (“Prodigiosin biosynthesis,” “Tropane, piperidine and pyridine alkaloid biosynthesis,” “Caprolactam degradation,” “Nitrogen metabolism,” “Biosynthesis of unsaturated fatty acids”), and one of which was related to Environmental Information Processing (“ABC transporters”).

### TDGs were tissue-specific under salt stress

**Figure 4:**
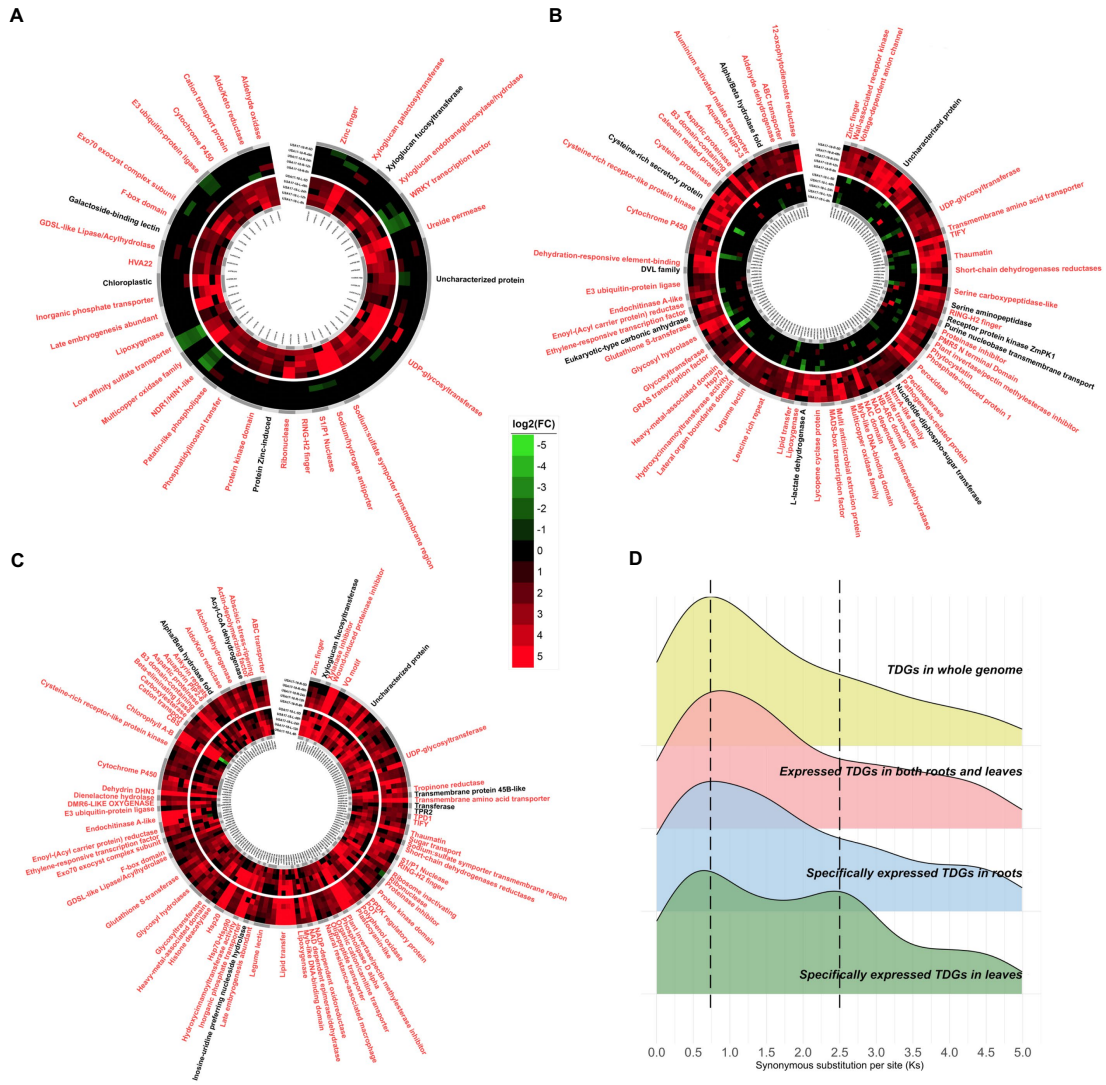
Tissue-specific expression of TDGs under salt stress. **(A)** Specifically expressed TDGs in leaves. Genes related to salt resistance with reviewed evidence were marked in red. **(B)** Specifically expressed TDGs in roots. **(C)** Expressed TDGs in both roots and leaves. **(D)** Distribution of Ks with different specific expression TDGs.

Time-ordered, comparative, transcriptome analyses were performed, which focused on the expression of TDGs under salt stress. A total of 302 TDGs were identified as having up-regulated expression in response to salt stress (Supplementary Table 7), of which 48 were specifically expressed in leaves (Figure 4A); 116 were specifically expressed in roots (Figure 4B), and 138 were co-expressed in both roots and leaves (Figure 4C). These genes were annotated according to the NR, Swissprot and Pfam databases, and then literature searches were conducted to confirm whether their functions were related to salt resistance. It was found that most TDGs appeared to respond to salt stress. Some of these genes had been annotated as “Uncharacterized protein” (Supplementary Table 7), and it can be speculated that these genes also play an important role in the adaptation of *P. vaginatum* to salt stress. Even in the same gene family, some family members were expressed only in leaves but others in roots, such as “Cytochrome P450,” “UDP glycosyltransferase” and “Multicopper oxidase family” etc.

The frequencies of synonymous substitution (Ks) were calculated to estimate the age of duplication events. There were similar peaks (~0.7) among the tissue-specifically expressed TDGs and the TDGs in whole genome. However, there was another peak (~2.5) with only tissue-specifically-expressed TDGs in leaves (Figure 4D), which included the gene functions for “Cation transporter,” “Sulfate transporter,” and “UDP-glycosyltransferase” etc. (Table 2).

**Table 2:** Specifically expressed TDGs in leaves with Ks of about 2.5.

Gene 1	Gene 2	Ka	Ks	Ka/Ks	Description
emOS121.100	emOS121.99	0.34	2.32	0.15	Galactoside-binding lectin
emFS28.27	emFS28.24	0.53	2.32	0.23	Uncharacterized protein
emOS169.431	emOS169.432	0.54	2.34	0.23	Protein Zinc-induced
emOS80.43	emOS80.44	0.53	2.43	0.22	E3 ubiquitin-protein ligase
emOS55.84.1	emOS55.83	0.46	2.49	0.18	Low affinity sulfate transporter
emFS4.55	emFS4.54	0.48	2.54	0.19	Cation transport protein
emFS28.24	emFS28.23	0.41	2.65	0.15	Uncharacterized protein
emFS26.201	emFS26.202	0.50	2.67	0.19	UDP-glycosyltransferase
emOS99.330	emOS99.331	0.26	2.84	0.09	Zinc finger
emOS56.23	emOS56.24	0.57	2.91	0.20	NDR1/HIN1-like
emOS36.477	emOS36.478	0.49	2.93	0.17	Xyloglucan fucosyltransferase
emOS55.83	emOS55.82	0.24	2.99	0.08	Low affinity sulfate transporter

### Analysis of ABC gene family in *Paspalum vaginatum*

**Figure 5:**
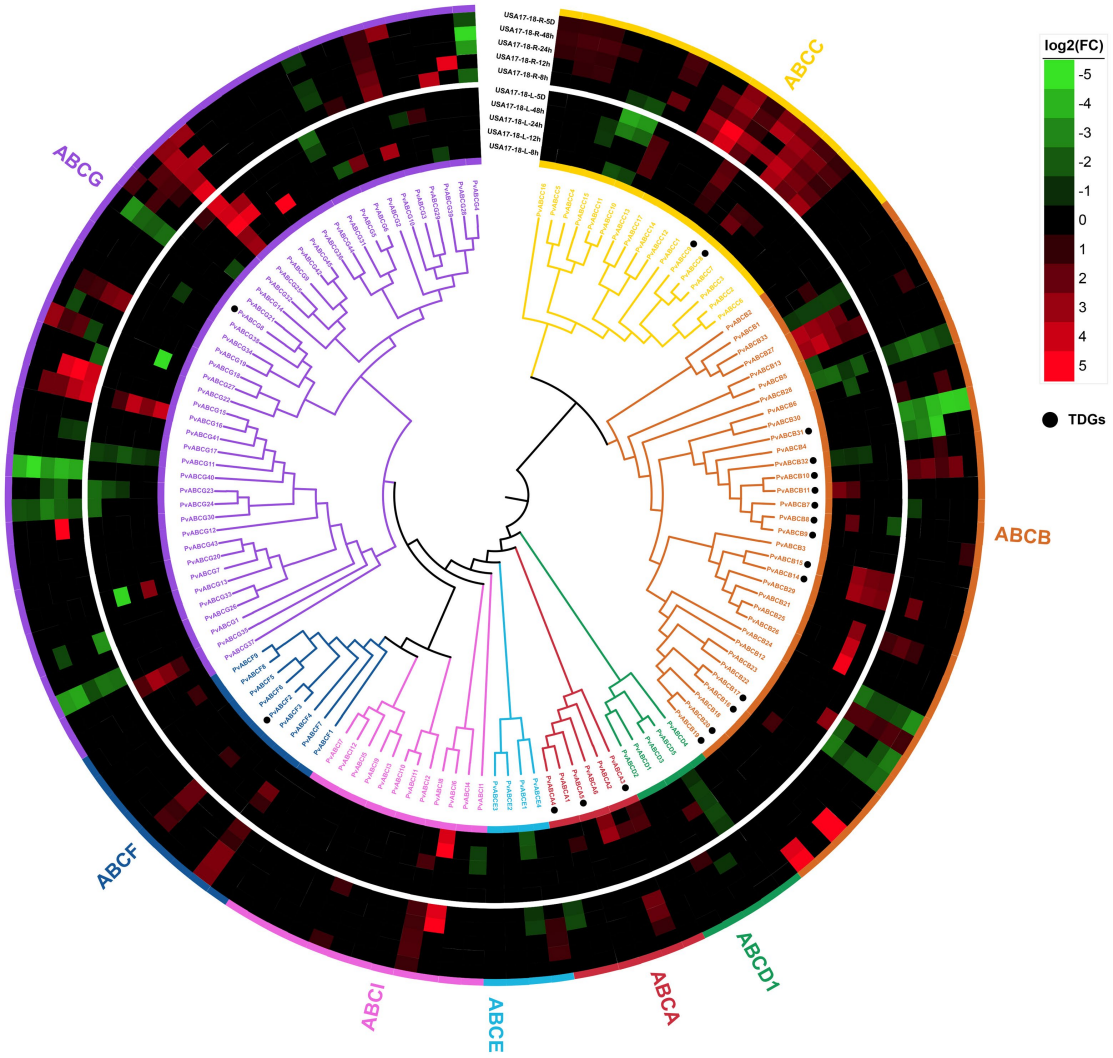
Phylogenetic tree of ABC transporter proteins from *P. vaginatum*. Eight subfamilies are highlighted in different colors. The genes marked with black dots represent TDGs. Heatmap shows transcriptome expression under salt stress.

A total of 131 *P. vaginatum* ABC protein sequences were identified. To further understand the relationship between the ABC family genes, full-length protein sequences of *P. vaginatum* and *O. sativa* ABC proteins were aligned to construct a phylogenetic tree. The result showed that the ABC genes of these two species can be divided into eight subfamilies (Supplementary Figure 1). The ABC family genes in *P. vaginatum* were denoted as *PvABCA1*-*PvABCA6*, *PvABCB1*-*PvABCB33*, *PvABCC1*-*PvABCC17*, *PvABCD1*-*PvABCD5*, *PvABCE1*-*PvABCE4*, *PvABCF1*-*PvABCF9*, *PvABCG1*-*PvABCG45*, and *PvABCI1*-*PvABCI12*, according to their chromosomal location (Figure 5). Among these subfamilies, PvABCG had the largest number of members with 45 genes; PvABCB was the second largest subgroup, containing 33 genes; and the smallest subgroup was PvABCE, containing only 4 genes. Molecular masses ranged from 15.81 kDa to 596.82 kDa and pIs ranged from 4.81 to 9.87 (Supplementary Table 8).

To better understand the biological functions of the ABC family genes, the combined *P. vaginatum* transcriptome under salt stress was analyzed for expression patterns. As shown in Figure 5, the *PvABCs* exhibited different expression levels under salt stress. *PvABCB14* and *PvABCB15*, identified as TDGs, were highly upregulated in roots, whereas *PvABCC8* and *PvABCC9*, also identified as TDGs, were highly upregulated in leaves under salt stress. Many *PvABCs* were not differentially expressed.

## Discussion

### Long-tandem clusters in *Paspalum vaginatum* are strongly associated with abiotic stress

With long-tandem clusters (more than six genes in the same cluster), chromosomes 3 and 6 had the largest numbers of long clusters (four and three, respectively). A total of 14 long-tandem clusters (containing 97 TDGs) were found to be associated with abiotic stress, with 12 specifically associated with salt stress (Table 1). Two clusters in particular (with nine and eight genes in each cluster) with function “UDP-glucosyl transferase” were of particular interest. Further research showed that “UDP-glucosyl transferase” can be associated with elevated antioxidant enzyme activity and reduced production of reactive oxygen species, which could control the oxidative burst under stress situations (Wang et al., 2021a). In the present study, many “UDP-glucosyl transferase” genes were identified resulting from tandem-duplication events, which have enriched the antioxidant capacity of *P. vaginatum*.

### The unique GO terms and KEGG pathways of TDGs in *Paspalum vaginatum* seem to indicate contributions to salt tolerance

GO and KEGG enrichment analyses were used to determine the possible roles of TDGs in *P. vaginatum*, and were also compared in four related species (*O. sativa*, *S. italica*, *S. bicolor* and *Z. mays*). It was discovered that *P. vaginatum* TDGs were associated with some unique GO terms (such as “ion transmembrane transporter activity,” “anion transmembrane transporter activity” and “cation transmembrane transport”). These GO terms indicate functions promoting osmoregulation, for example by accumulating compatible solutes to avoid osmotic stress caused by salinity (Kumari et al., 2015; Rahman et al., 2021). *P. vaginatum* TDGs were also found to be associated with some unique KEGG pathways, and one being “ABC transport.” ABC transporters belong to a large protein family that utilize the energy released by ATP hydrolysis to transport a wide range of substrates across biological membranes. ABC transporters are involved in diverse cellular processes such as biotic and abiotic stress responses through plant hormone transport, ion transport, lipid transport and redox homeostasis (Do et al., 2018; Dahuja et al., 2021). In the present study, 131 ABC transporter genes were identified in *P. vaginatum*, with 13 (40%) of the 33 genes identified being TDGs. PvABCB was the second largest subgroup in the ABC transporter family and contained the most TDGs. *PvABCB14* and *PvABCB15*, identified from TDGs, were highly upregulated in roots, which might have an important role in enhancing the salt resistance of *P. vaginatum*.

### The evolution of roots and leaves in response to salt stress

With transcriptome expression available at multiple time points under salt stress, *P. vaginatum* TDGs were divided into those with leaf-specific expression (48 TDGs), root-specific expression (116 TDGs), or co-expression in both leaves and roots (138 TDGs). Compared to leaves, roots had more TDGs in response to salt stress, possibly indicating that roots are more important than leaves in adapting to saline environments. Interestingly, when Ks was calculated for tissue-specifically expressed TDGs and compared with that for TDGs in whole genome, a similar peak (~0.7) was found, whereas another peak (~2.5) was found only in tissue-specifically expressed TDGs in leaves. Therefore, it was speculated that leaves became adapted to salt stress in an earlier whole genome duplication event (WGD; ~83.3 million years ago; Ma), whereas the entire *P. vaginatum* plant acquired a large number of TDGs related to salt stress in a second WGD (~23.3 Ma). Annotations of these expressed TDGs were found from multiple databases and it was found that most were related to salt stress. We therefore speculate that expressed TDGs annotated as “Uncharacterized protein” also contribute to salt resistance in *P. vaginatum*, and these genes could serve as potential and novel genes related to salt resistance.

### Subfunctionalization of tandem duplicated pairs

Duplicate genes can diverge in function, representing a potential source of response mechanisms to survive stressful environments (Zou et al., 2009; Arsovski et al., 2015) and the TDGs in *P. vaginatum* have shown functional divergence. For example, emFS26.201 and emFS26.202 are tandem duplicated pairs (annotated as “UDP-glycosyltransferase”), but emFS26.201 has leaf-specific expression while emFS26.202 has root-specific expression under salt stress. As another example, from TDGs in the same tandem cluster, emOS168.286, emOS168.288, emOS168.289, and emOS168.290 (annotated as “Peroxidase”), only emOS168.290 responds to salt stress (Supplementary Table 9). A total of 302 TDGs were identified in response to salt stress, and these TDGs were part of tandem clusters comprising 623 TDGs (Supplementary Table 9). In other words, nearly half of these duplicate lineages retain stress responsiveness.

### The role of TDGs in evolution and adaptation

Evolutionary innovation is often built on variations from redundant genetic materials generated by gene duplication, and tandem duplication represents a potential source for such innovation (Arsovski et al., 2015; Guo et al., 2019). Previous studies have shown that: TDGs have driven diversification of the protein modifier SUMO in angiosperms (Hammoudi et al., 2016); TDGs have driven the divergent evolution of caffeine and crocin biosynthetic pathways in *Gardenia jasminoides* (Xu et al., 2020); TDGs are involved in defense and pollinator attraction in *Tectona grandis* (Zhao et al., 2019); and lastly that TDGs are significantly enriched in resistance-related pathways and more abundant in retrotransposon-related genes in *Cajanus cajan* (Liu et al., 2021). In this study, we identified TDGs in the *P. vaginatum* and found that TDGs of *P. vaginatum* were enriched in many unique GO terms and KEGG pathways which were associated with resistance, especially salt resistance. The TDGs associated with response to salt stress were identified by transcriptome analyses at multiple time points. This study provides insights into the roles of tandem duplications in the evolution and adaptation of *P. vaginatum* and lays the foundation for the genomics-based breeding of other grasses.

## Data availability statement

The datasets presented in this study can be found in online repositories. The names of the repository/repositories and accession number(s) can be found in the article/Supplementary material. RNA-Seq data obtained have been uploaded to the National Center for Biotechnology Information (https://www.ncbi.nlm.nih.gov/) and can be accessed with accession number PRJNA874860.

## Author contributions

XH and J-SH contributed equally to this work performed data analyses, and wrote the manuscript. Z-YW and LL conceived and managed the project. MQ-T and YC designed the experiments. TX and L-ZR identified genes related to salt resistance through literature search. LP interpreted the results and revised the manuscript. All authors contributed to the article and approved the submitted version.

## Funding

This work was supported by the National Natural Science Foundation of China (No.32060409), Key scientific research projects of colleges and universities in Hainan Province (No. Hnky2019ZD-3), the Construction of World First Class Discipline of Hainan University (No.RZZX201905), and National Project on Sci-Tec Foundation Resources Survey (2017FY100600).

## Conflict of interest

The authors declare that the research was conducted in the absence of any commercial or financial relationships that could be construed as a potential conflict of interest.

## Publisher’s note

All claims expressed in this article are solely those of the authors and do not necessarily represent those of their affiliated organizations, or those of the publisher, the editors and the reviewers. Any product that may be evaluated in this article, or claim that may be made by its manufacturer, is not guaranteed or endorsed by the publisher.

## Footnotes

^1^ http://plants.ensembl.org/index.html

^2^ http://eggnog-mapper.embl.de/

^3^ http://pfam.xfam.org/

^4^ https://www.ncbi.nlm.nih.gov/cdd

^5^ https://web.expasy.org/protparam/

^6^ https://itol.embl.de/

## Supplementary material

SUPPLEMENTARY FIGURE 1 Phylogenetic tree of ABC transporter proteins from *P. vaginatum and O. sativa*. Eight subfamilies are highlighted in different colors.

SUPPLEMENTARY TABLE 1 Statistics of TDGs in *P. vaginatum* and other studied species.

SUPPLEMENTARY TABLE 2 Number of TDGs in chromosome of *P. vaginatum* genome.

SUPPLEMENTARY TABLE 3 Calculation of synonymous and nonsynonymous substitutions per site of TDG pairs.

SUPPLEMENTARY TABLE 4 TDGs with more than six genes in the same cluster and their functions.

SUPPLEMENTARY TABLE 5 GO enrichment results of TDGs in *P. vaginatum*.

SUPPLEMENTARY TABLE 6 KEGG enrichment results of TDGs in *P. vaginatum*.

SUPPLEMENTARY TABLE 7 TDGs which response to salt stress in *P. vaginatum.*

SUPPLEMENTARY TABLE 8 Basic information about the ABC transporter gene family in *P. vaginatum*.

SUPPLEMENTARY TABLE 9 Information of tandem clusters containing 302 tissue-specific TDGs.

SUPPLEMENTARY TABLE 10 Quality control of RNA-seq.

SUPPLEMENTARY TABLE 11 Expression (FPKM) of genes in *P. vaginatum* under salt stress.
